# Comprehensive high-throughput image analysis for therapeutic efficacy of architecturally complex heterotypic organoids

**DOI:** 10.1038/s41598-017-16622-9

**Published:** 2017-11-30

**Authors:** Anne-Laure Bulin, Mans Broekgaarden, Tayyaba Hasan

**Affiliations:** Wellman Center for Photomedicine, Department of Dermatology, Harvard Medical School and Massachusetts General Hospital, 40 Blossom Street, 02114 Boston, MA USA

## Abstract

Bioengineered three-dimensional (3D) tumor models that incorporate heterotypic cellular communication are gaining interest as they can recapitulate key features regarding the intrinsic heterogeneity of cancer tissues. However, the architectural complexity and heterogeneous contents associated with these models pose a challenge for toxicological assays to accurately report treatment outcomes. To address this issue, we describe a comprehensive image analysis procedure for structurally complex organotypic cultures (CALYPSO) applied to fluorescence-based assays to extract multiparametric readouts of treatment effects for heterotypic tumor cultures that enables advanced analyses. The capacity of this approach is exemplified on various 3D models including adherent/suspension, mono-/heterocellular cultures and several disease types. The subsequent analysis revealed specific morphological effects of oxaliplatin chemotherapy, radiotherapy, and photodynamic therapy. The procedure can be readily implemented in most laboratories to facilitate high-throughput toxicological screening of pharmaceutical agents and treatment regimens on organotypic cultures of human disease to expedite drug and therapy development.

## Introduction

Recent reports have illustrated that the complexity of cancer tissues composed of various populations of malignant and supporting cell types set in a dynamic microenvironment^1,2^, gives rise to high levels of intrinsic heterogeneity, architectural complexity, and treatment-resistant phenotypes in tumor masses^3–5^. Therefore, the traditional two-dimensional (2D) cell culture models provide limited insights into tumor biology and the development of new cancer therapeutics. In this context, three-dimensional (3D) organotypic cultures are emerging as highly useful *in vitro* models for a multitude of cancer types that faithfully recapitulate various key features of the clinical presentation of these complex diseases^6–12^. These 3D models bridge a gap between highly relevant but low throughput *in vivo* animal models and the high throughput 2D cultures with low clinical relevance, and thus hold promise as representative initial testing platforms of therapeutics and expedite their development.

However, although there is a growing appreciation for organotypic models of human disease and increasing varieties of 3D culture methods, the implementation of 3D cultures as a mainstream approach for expedited therapy screening requires the development or adaptation of quantitative analysis methods^7^. Indeed, there remains a relative scarcity in assays that provide functional and reliable readouts for therapeutic drug screening and assessment of treatment outcomes. The necessity for these assays is further underscored by the limitations of colorimetric toxicity assays typically used for 2D cultures (*e*.*g*., tetrazolium and trypan blue assays) that are not optimized for 3D culture models^13^, and which simplify treatment responses by reporting a single metric that may not accurately reflect the full scope of effects^14^. For 3D cultures, live/dead staining utilizing calcein AM and propidium iodide (PI)/ethidium bromide/DRAQ7/DAPI are widely used for qualitative purpose^15–17^ but are not frequently used as a quantitative readout. To address this scarcity in information-rich treatment response assays for organotypic cultures, several groups have developed useful imaging-based platforms to investigate tumor architecture and viability^14,18^. These assays derive volumetric readouts from single-plane fluorescence or brightfield images, rendering their application appropriate for spherical organoids, but not for more complex architectural structures^18^.

**Figure 1:**
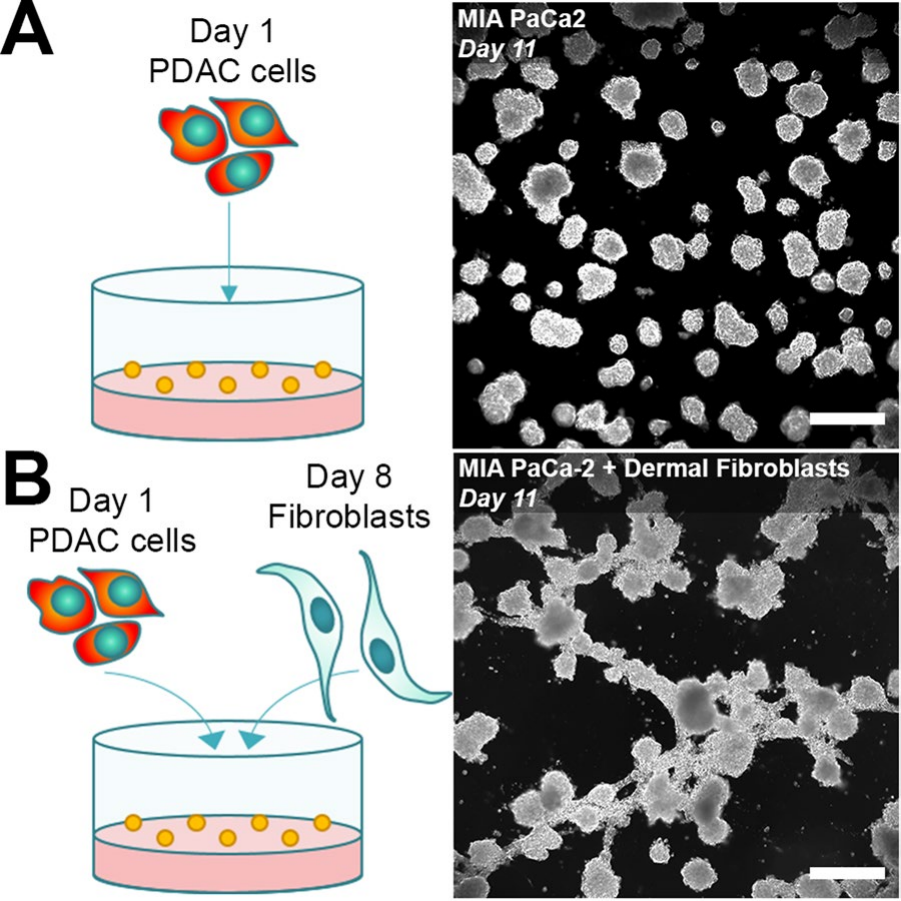
Heterotypic 3D cultures generate a heterogeneous mix of tumors with architecturally complex geometries. (**A**) A conventional monoculture of PDAC cells (MIA PaCa-2 cell line) develop into asymmetrical spheroids over 11 days. (**B**) When MIA PaCa-2 cells are cocultured with fibroblasts (primary dermal fibroblast cell line), a heterogeneous mix of non-spheroidal organoids are formed. Images were taken using darkfield microscopy. (Scale bar = 500 µm).

As the heterocellular and heterogeneous nature of cancer tissues appears to be a key feature that drives treatment response^19–22^, heterotypic tumor organoid models with intricate non-spheroidal architectures that recapitulate stromal features become more frequently investigated^23–28^. When grown in direct coculture with stromal cells, tumor organoids with severely heterogeneous and complex architectures are formed (Fig. 1). This complexity renders volumetric analyses of treatment response, in which spherical shapes are assumed, inaccurate. There is currently no established method to assay the sophisticated array of potential treatment responses in such architecturally complex organoid cultures. As 3D cultures with increased complexity are more routinely used, it is essential that the analysis methods evolve at the same pace and that advancements in computational tools should be leveraged to facilitate this development.

To address this challenge, we outline a high-throughput image analysis procedure of fluorescence microscopy images that allows comprehensive *in situ* assessment of treatment responses on 3D cancer cultures with intricate non-spherical geometries. Hereon, we will refer to these tumor nodules as organoids, and the image analysis procedure as *CALYPSO*; **C**omprehensive image **A**na**LY**sis **P**rocedures for **S**tructurally complex **O**rganotypic cultures. This method leverages a well-established live/dead staining protocol of the cultures that utilizes calcein AM and PI stains for live and dead cells respectively^29^, and the subsequent fluorescence image analysis recapitulates the experimental strengths of the procedure described by *Celli et al*.^14^. The calcein AM is retained by live cells through cleavage of its ester bonds by intracellular esterases yielding a green-fluorescent cytoplasmic calcein, while PI selectively binds the DNA of necrotic cells as it is incapable of crossing the plasma membrane of viable cells.

However, CALYPSO utilizes a new approach in terms of image processing to extract the functional parameters on a nodule-by-nodule basis, recording multiple readouts for individual organoids that enables advanced assessments of treatment response. This methodology disregards differences in treatment susceptibility between cell subpopulations within the organotypic cultures, yet considers the tumor organoids as a whole, which is comparable to how treatment responses are monitored in patients. We demonstrate the capacity of CALYPSO to extract multiparametric readouts from various 3D culture methods and disease types as exemplified on (1) adherent and (2) suspended 3D cultures of pancreatic cancer, as well as (3) an adherent 3D culture model of micrometastatic ovarian carcinoma. The methodology can furthermore be applied to various treatment types, which we illustrate for (1) oxaliplatin chemotherapy, (2) X-ray radiation therapy, and (3) photodynamic therapy. In addition, we provide elaborate proof-of-concept and propositions for advanced data analysis that is not typically available using conventional colorimetric/luminescent assays of viability. Importantly, the results in this study were obtained with commercially available materials and imaging systems that are widely available, rendering the platform highly feasible for implementation in most laboratories. Although the methodology is exemplified on a limited number of data sets, it can potentially be applied to other organotypic cultures, treatments, and can easily be adapted to suit other fluorescence-based assays.

## Results and Discussion

### Establishing the image analysis workflow

In order to provide a robust treatment response assay for architecturally complex 3D tumor models, we established an image analysis workflow that enables the extraction of functional area- and fluorescence intensity-based parameters. The experimental procedure to derive treatment response data leverages an established fluorescence based live/dead assay that consists of staining viable and necrotic cells, respectively. The staining of the organotypic cultures *in situ* is performed using calcein AM and PI^14,29^. Conventional confocal fluorescence microscopy is then performed at low magnification to record the fluorescence intensities of both the live and dead stains, alongside a corresponding brightfield image of the culture. These three image types serve as the basis for the subsequent image analysis workflow to enable comprehensive evaluation of the individual tumor organoids within the 3D cultures. As a rule of thumb, all experiments require no treatment and total killing control groups to accurately set the dynamic range of the acquisition parameters and analysis procedures.

The image analysis workflow (CALYPSO) outlined in this communication leverages the strength of the live/dead staining protocol previously described by *Morris et al*., and more recently by *Celli et al*.^14,29^, yet utilizes a new approach to extract quantitative data from the fluorescence and brightfield images. Moreover, CALYPSO accurately determines the organoid area regardless of shape rather than relying on imprecise volumetric estimations for such nodules. The cornerstone of our analysis is the identification and indexation of every individual tumor organoid based on the brightfield images. However, as non-uniform illumination results in various degrees of brightness in the brightfield image, binarizing the images using a constant threshold value would prohibit correct identification of the tumor nodules. To overcome this limitation, CALYPSO employs adaptive thresholding, which calculates a local threshold for every pixel within the image and binarizes the brightfield image using these local threshold values to accurately detect the outline of the individual organoids. A size threshold of 1931µm^2^ (50px) identifies objects of an area equivalent to approximately five clustered cells to be a tumor organoid, and excludes smaller objects such as culture debris or individual migrating cells from subsequent analyses. A second filtering step is added to the masking process that excludes objects that are out-of-focus from the masks. To do so, the fluorescence channels are summed and the resulting images are subsequently binarized using a threshold calculated with Otsu’s method^30^. This binary image is then multiplied with the previously obtained mask to exclude the organoids that are out-of-focus. Using the final mask, each individual organoid is indexed and because this index is kept unique throughout the full analysis procedure, all subsequent parameters are given in a nodule-by-nodule fashion. Amongst these parameters are the *total area*, *viable area*, and *viability* of the individual organoids, which are calculated as described below.

Additional quantitative information is derived from analyzing the fluorescence intensities of calcein and PI. First, fluorescence signals need to be corrected for the background intensity. The most reliable way to extract the background was by using the inter-organoid space, isolated by inversing the mask. However, given that many bright fluorescent loci (e.g., individual detached cells, cell debris) were present for both fluorophores within the inverse mask, calculating the average background intensity of the inverse mask for each image typically resulted in overcorrection. We found that the most appropriate approach consisted of determining the median background intensities for the calcein AM (live) and PI (dead) fluorescence on images from the no treatment group. The organoids in these wells were typically intact and were measured using identical experimental parameters as the treated groups within every experiment. The median live and dead fluorescence intensities were averaged over multiple inverse masks to obtain accurate background values for both channels, which were subsequently subtracted from every image. Afterwards, CALYPSO determines the viability of each individual tumor nodule, using the ratio defined in (1).

1 $$Viability=\frac{Intensit{y}_{CalceinAM}}{Intensit{y}_{CalceinAM}+Intensit{y}_{PI}}$$

The viability reports the health of the individual tumor organoids that varied between 0 and 1 as the total killing and no treatment control groups were used to set the experimental fluorescence acquisition parameters. An additional metric, in the form of viable tumor area, can be calculated to quantify the remaining viable disease. Unlike the viability calculations that rely on the fluorescence intensities, analysis of the live area requires binarization of the images to distinguish between viable and non-viable fractions of the tumor organoids. We found that the live threshold value is reliably estimated by applying Otsu’s method^30^ and establishing the dynamic range utilizing the no treatment (total area equals live area) and total killing (total area has no live area) control groups. After binarization, the absolute live area of the residual disease (in µm^2^) is quantified, as well as the fractional live area for each indexed organoid, as defined in (2).

2 $$Frac.\,live\,area=\frac{Live\,area}{Total\,area}$$

**Figure 2:**
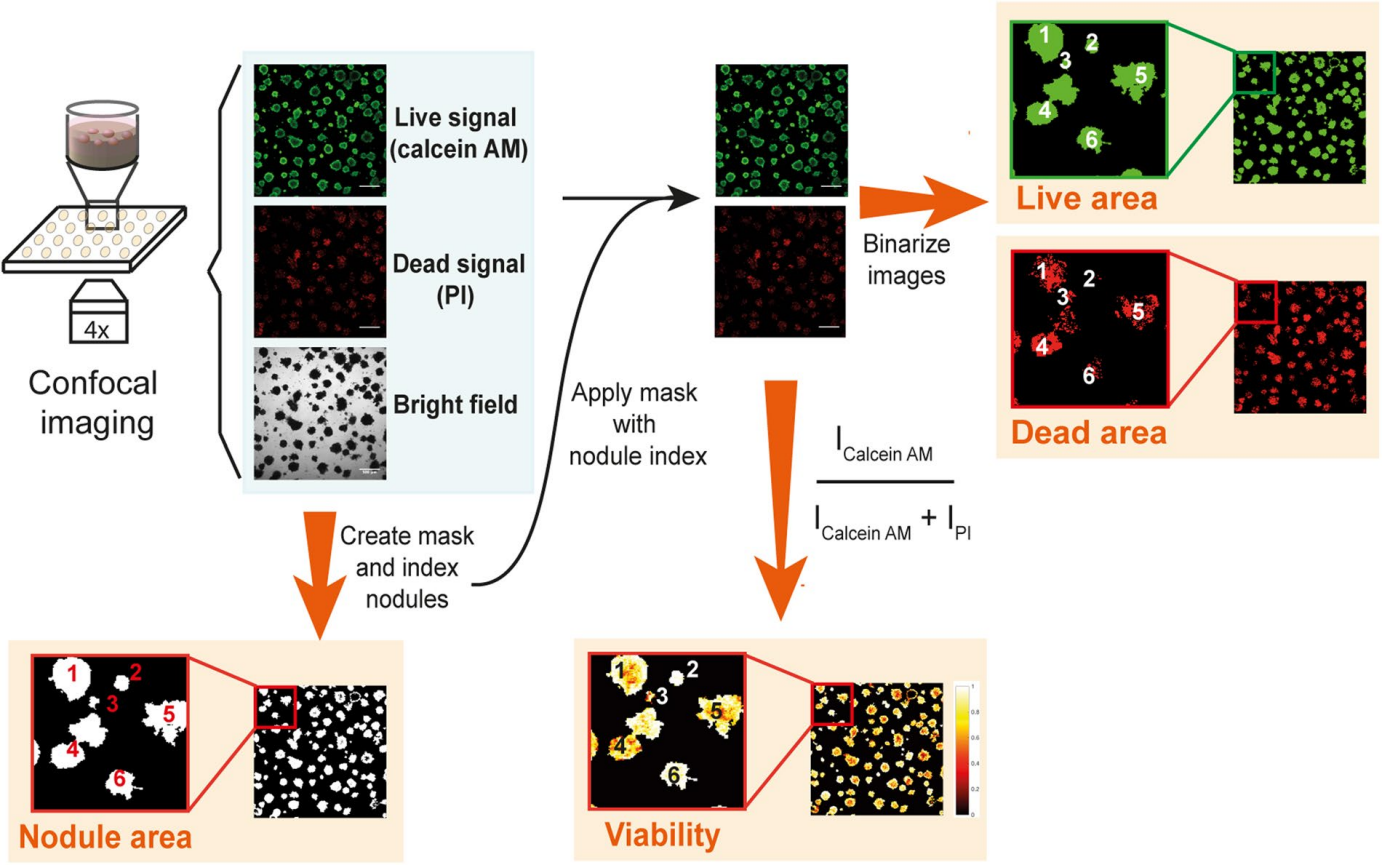
Schematic representation of the image analysis workflow following live/dead staining and confocal fluorescence imaging of 3D cultures. Following acquisition of the live and dead fluorescence and brightfield images, a mask is created from the brightfield images and individual objects (*i*.*e*., organoids) are indexed. The mask is then applied to both fluorescence images, after which the intensities per object are extracted for subsequent viability calculations. The masked fluorescence images are then thresholded to extract the live and dead area for every object. This method links all readouts are directly linked to the object index.

A similar binarization approach can be applied to the dead channel, yielding the absolute dead area and fractional dead area for every indexed organoid, although this is not further explored in this study. Through these procedures, CALYPSO enables the analysis of the size, health (viability), and extent of viable tissue on 3D cultures with both spherical and more complex non-spherical geometries for every individual tumor organoid within the 3D culture, thus allowing comprehensive multiparametric analysis of treatment response dynamics. A global schematic of the CALYPSO workflow is depicted in Fig. 2.

### Validation of the methodology for analyzing various cancer treatment effects

To demonstrate the capacity of CALYPSO to extract the treatment response readouts in a reliable manner, we established a 3D adherent culture of MIA PaCa-2 pancreatic cancer cells (Fig. 1A). Pancreatic cancer was chosen as current treatment modalities yield unsatisfactory clinical results, and 3D models may thus represent a promising therapy screening platform^31^. In addition to the no treatment and total killing control groups, three mechanistically different cancer therapies were applied to these cultures. We subjected the pancreatic organoids to photodynamic therapy (PDT)^32^, oxaliplatin chemotherapy^33^, and X-ray radiotherapy (RT)^34^, and combinations thereof. Those therapeutic modalities are all clinically used to address pancreatic ductal adenocarcinoma (PDAC)^32–34^, a lethal disease presenting a high intrinsic treatment resistance that largely attributed to its dense stromal compartment^35^. A brief description on the rationale and cytostatic mechanisms is provided below.

Photodynamic therapy (PDT) is a cancer treatment that utilizes a light-absorbing pharmaceutical (photosensitizers) to induce cytotoxicity within the tumor tissue. Upon irradiation of the tumor with light suitable to excite the photosensitizer, the photochemical production of cytotoxic reactive oxygen species causes massive tumor cell death through three mechanisms. While direct tumor cell death is achieved through oxidation of vital biomolecules and biomembranes and induces an anti-tumor immune response, tumor vasculature shutdown leads to hypoxia and hyponutrition^36–38^. PDT is widely applied in the treatment of dermal afflictions and is being explored as an adjuvant and palliative treatment for various type of terminal cancers^39–41^. For PDAC, a recent clinical trial demonstrated that PDT with the photosensitizer benzoporphyrin-derivative (BPD) was feasible and safe, and showed promise in reducing tumor burdens so that non-resectable patients became eligible for curative surgery^32^.

Oxaliplatin chemotherapy is an essential component of the FOLFIRINOX chemotherapy regimen, which is considered an effective albeit highly toxic strategy to treat inoperable pancreatic cancer. FOLFIRINOX achieves median patient survival of 24.2 months, compared to 7.4 months with gemcitabine, the standard of care^33,34^. Mechanistically, oxaliplatin crosslinks DNA, thereby prohibiting cell DNA replication, cell division, and promoting apoptotic cell death via a DNA damage response^42^.

The use of radiation therapy (RT), which induces DNA damage through irreparable base oxidation by radiation-induced free radical species^43^, remains controversial for PDAC. Numerous clinical trials have been performed and no consensus has yet been reached regarding its efficacy. However, when used in combination with a chemotherapy agent, chemoradiotherapy showed a significant improvement in the progression-free survival (10.8 months vs 7.4 months) and in the overall survival (15 months vs 11.7 months) in patients that first received 3 months of gemcitabine^44^.

**Figure 3:**
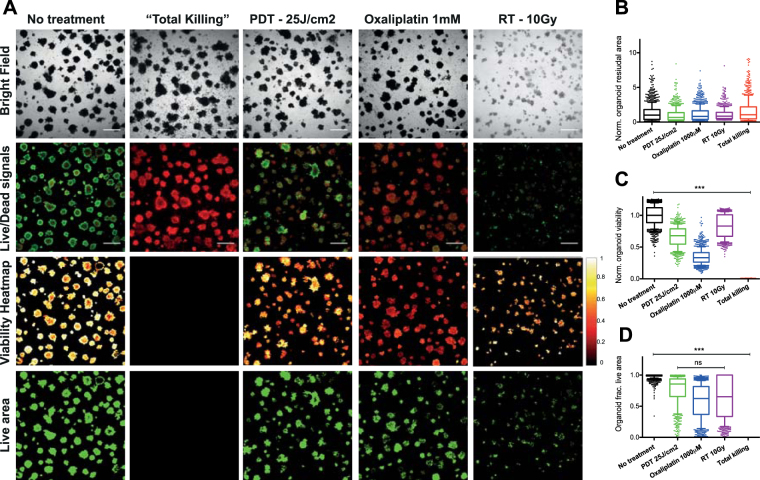
Treatment outcomes of PDAC 3D models grown as adherent monocultures (MIA-PaCa2) subjected to PDT (25 J/cm^2^), oxaliplatin chemotherapy (1 mM) and radiotherapy (10 Gy). (**A**) Brightfield and live/dead fluorescence images, obtained using a confocal microscope, as well as viability heatmaps and live area maps derived from subsequent CALYPSO analysis are given for each group. (**B**) Boxplot depicting the spread in normalized organoid total areas as quantified from the brightfield images depicted in panel A. (**C**) Boxplot depicting the spread in viability of every individual organoid within the treatment and control groups. (**D**) Boxplot depicting the spread in fractional live area of every individual organoid within the treatment and control groups. All boxplots depict median, 25^th^ and 75^th^ percentile, and the 90% confidence interval.

In Fig. 3, the effects of the above-mentioned treatments on 3D PDAC cultures are shown. The no treatment control groups demonstrated high viability with individual tumor nodules displaying a core-shell structure with lower viability in the center, a characteristic of hypoxic core formation^45^. The total killing wells showed intact tumor nodules in brightfield with PI fluorescence, yet display complete absence of calcein fluorescence. Consequently, the viability heatmap and the live area mask appear completely dark, indicating no viable organoids in the total killing controls. The most important notion is that the controls indicate correct image correction, processing, and thresholding of the live (calcein) fluorescence signal, thereby correctly defining the dynamic range of viability and fractional live area as quantitative metrics of treatment outcomes.

Following PDT or oxaliplatin chemotherapy, the brightfield images display equal distributions in tumor nodule size and quantity, which is reflected in the quantification of the total area (Fig. 3B). In contrast, the live/dead fluorescence images reveal that most nodules had substantially reduced calcein and increased PI fluorescence intensities. Viability heatmaps (Fig. 3A) and viability quantifications (Fig. 3C) confirm these observations. The residual live area panels suggest a substantially decreased residual viable disease for both treatments, which is reflected in the nodule-by-nodule analysis (Fig. 3D). However, despite observing no clear differences in overall viability between the PDT and oxaliplatin treatment groups, analysis of the live area suggested that most of individual tumor nodules remain viable following oxaliplatin chemotherapy, whereas a significantly lower fractional live area is observed in the PDT treated group (Fig. 3D). Lastly, the treatment effects of X-ray RT demonstrated a clear reduction in total organoid area on the brightfield images. Individual tumor nodules were smaller and had low contrast, suggesting thin plaques of clustered tumor cells. The live/dead fluorescence images, viability heatmaps, and viability quantification revealed that the residual tumor nodules had a relatively high viability and fractional live area (Fig. 3C), despite having a low cumulative residual live area.

Taken together, these results demonstrate the capacity of CALYPSO to perform adequate analysis of the brightfield and live/dead images for advanced examination of treatment effects. The analysis also provides compelling evidence that derivation of a single parameter will understate the full scope of treatment effects that may be effectuated by a specific cancer treatment and emphasizes the importance of the multifaceted approach described here.

### Validation of CALYPSO on architecturally complex cocultures of pancreatic cancer

**Figure 4:**
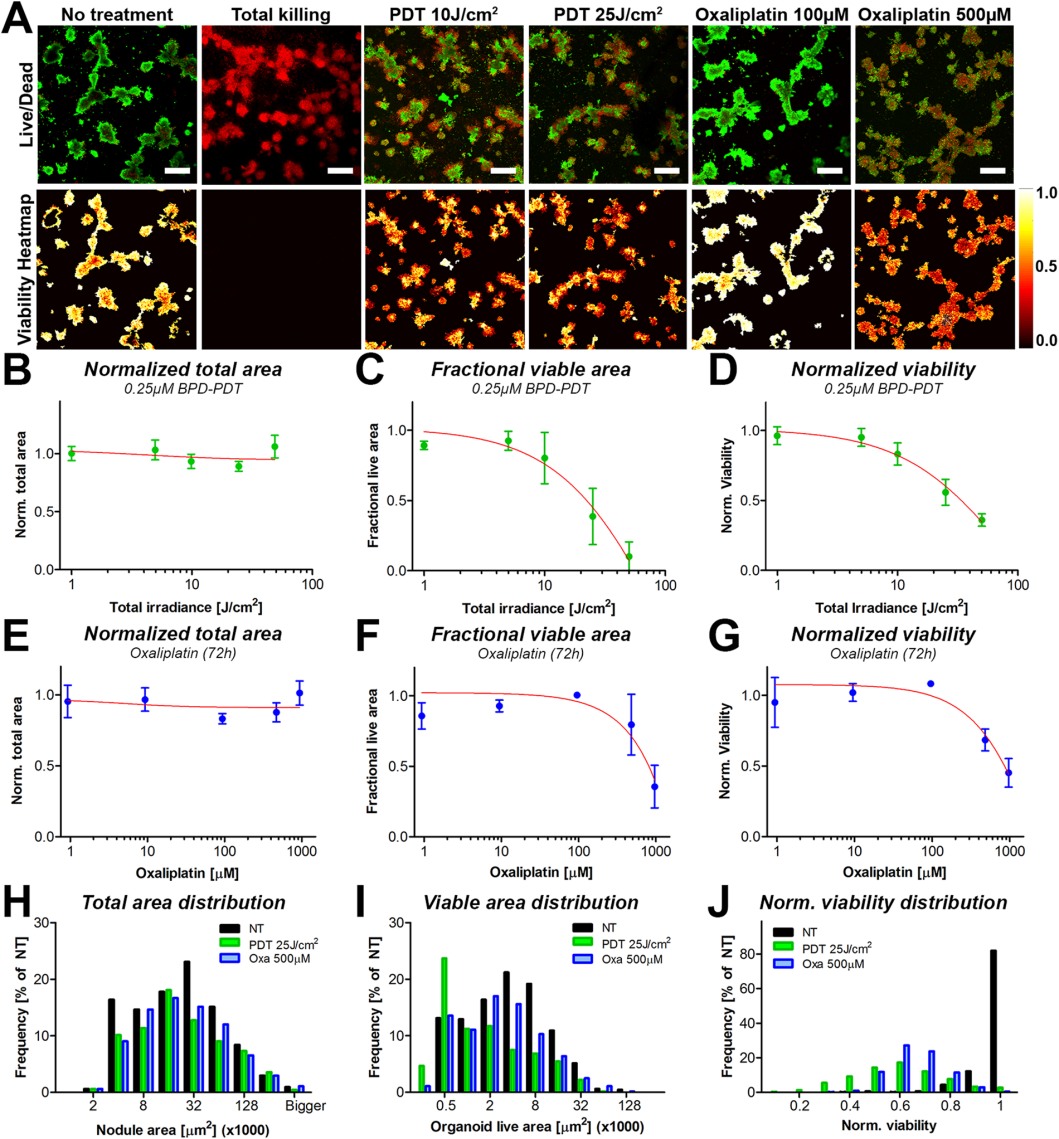
Primary output parameters obtained through CALYPSO, including viability heatmaps as well as normalized total area, fractional live area and normalized viability, demonstrate the ability to report treatment response dynamics on spheroid and non-spheroid organoids of MIA PaCa-2 cells grown with primary dermal fibroblasts following treatment with PDT and oxaliplatin chemotherapy. (**A**) Live/dead images of calcein and PI fluorescence were superimposed in ImageJ and depicted side-by-side with the corresponding viability heatmaps, providing spatial information on the viability distributions throughout the tumor nodules. Depicted are untreated adherent cocultures of MIA PaCa-2 cells and primary dermal fibroblasts, either untreated or treated with 25 J/cm^2^ BPD-PDT or 1 mM oxaliplatin (72 h). Dose response correlations between the PDT radiant exposure and the (**B**) median total area (mean ± SEM), (**C**) median fractional live area (mean ± st. dev.), and (**D**) the median viability of the tumor organoids (mean ± st. dev.). Data represents the mean of the median value per image (N = 12–24). (**E**–**G**) Distributions of residual total area (**E**), fractional live area (**F**) and viability (**G**) of the individual tumor organoids following treatment with BPD-PDT at a radiant exposure of 25 J/cm^2^ (green bars) or 500 μM oxaliplatin (blue bars) in comparison to the no treatment control group (black bars).

Recapitulating cancer heterogeneity requires the presence of different cell types and any analysis method needs to be able to incorporate this complexity. A major strength of CALYPSO is its ability to derive functional treatment response metrics from 3D cultures with intricate non-spherical architectures. To illustrate this capacity, we cocultured MIA PaCa-2 spheroids with primary dermal fibroblasts to yield organoids with a highly heterogeneous size distribution and non-spherical architectures (Figs 1B and 4A), and applied BPD-PDT and oxaliplatin chemotherapy to these cultures.

The dose-response dynamics of both treatments on the heterotypic PDAC organoids were extracted with CALYPSO and are summarized in Fig. 4. As expected from the previous results (Fig. 3), PDT and oxaliplatin minimally affected the total organoid area, as assessed by brightfield image analysis (Fig. 4B and E). The organoid fractional live area demonstrated a strong dose-response correlation for PDT (Fig. 4C), which was also observed upon analysis of the organoid viabilities albeit in a less dramatic manner (Fig. 4D). The dose-dependent reduction in organoid fractional live area was affected to a milder extent with oxaliplatin chemotherapy (Fig. 4F), and recapitulated the viability dose-response curve (Fig. 4G). To consider the intrinsic spread of the data, histograms of the total area distributions for both treatments in relation to the no treatment control group were plotted. A large spread in the organoid size and a slight reduction in absolute organoid numbers were observed, yet no major effect on the organoid total area distribution was reported as measured 96 h post-treatment (Fig. 4H). Whereas the no treatment group displayed similar distributions for the residual live area and total area, PDT decreased the number of viable organoids, and shifted their distribution towards small viable areas. Conversely, oxaliplatin chemotherapy reduced the total number of organoids, yet no shift towards smaller organoid live areas was observed (Fig. 4I). The distribution of viability scores indicates that for both treatments, the viability distribution was concise for oxaliplatin, but more widely spread for BPD-PDT (Fig. 4J). Together, the different metrics imply that PDT exerts its tumoricidal effects by reducing the size of the tumor organoids, thereby substantially reducing the residual viable disease, yet the remaining viable cores are of relatively high viability. Oxaliplatin treatment reduces the organoid viable area and viability more homogeneously compared to PDT, and reduced viability and live area regardless of organoid size. The quantitative data correlates well with the live/dead fluorescence images and the viability heatmaps (Fig. 4A), altogether illustrating the capacity of CALYPSO to derive dose-response relationships for various treatments.

To illustrate that CALYPSO also functions on 3D cultures grown under different conditions and following multiple combined treatments, the supplementary material includes a PDT-dose response analysis on an adherent culture of OVCAR-5 human ovarian carcinoma cells (Fig. S1), a PDT dose response analysis of AsPC-1 human pancreatic cancer cells grown as suspended spheroids (Fig. S2), and an analysis of an AsPC-1 adherent culture subjected to PDT combined with oxaliplatin chemotherapy (Fig. S3). In all cases, CALYPSO efficiently derived the readouts in a consistent and reproducible manner, underscoring that CALYPSO can be applied to multiple iterations of 3D cultures to extract informative readouts for different organoids, as well as various types of treatments and variations thereof.

### Correlations between organoid size, residual viable disease and viability informs the state of residual disease

**Figure 5:**
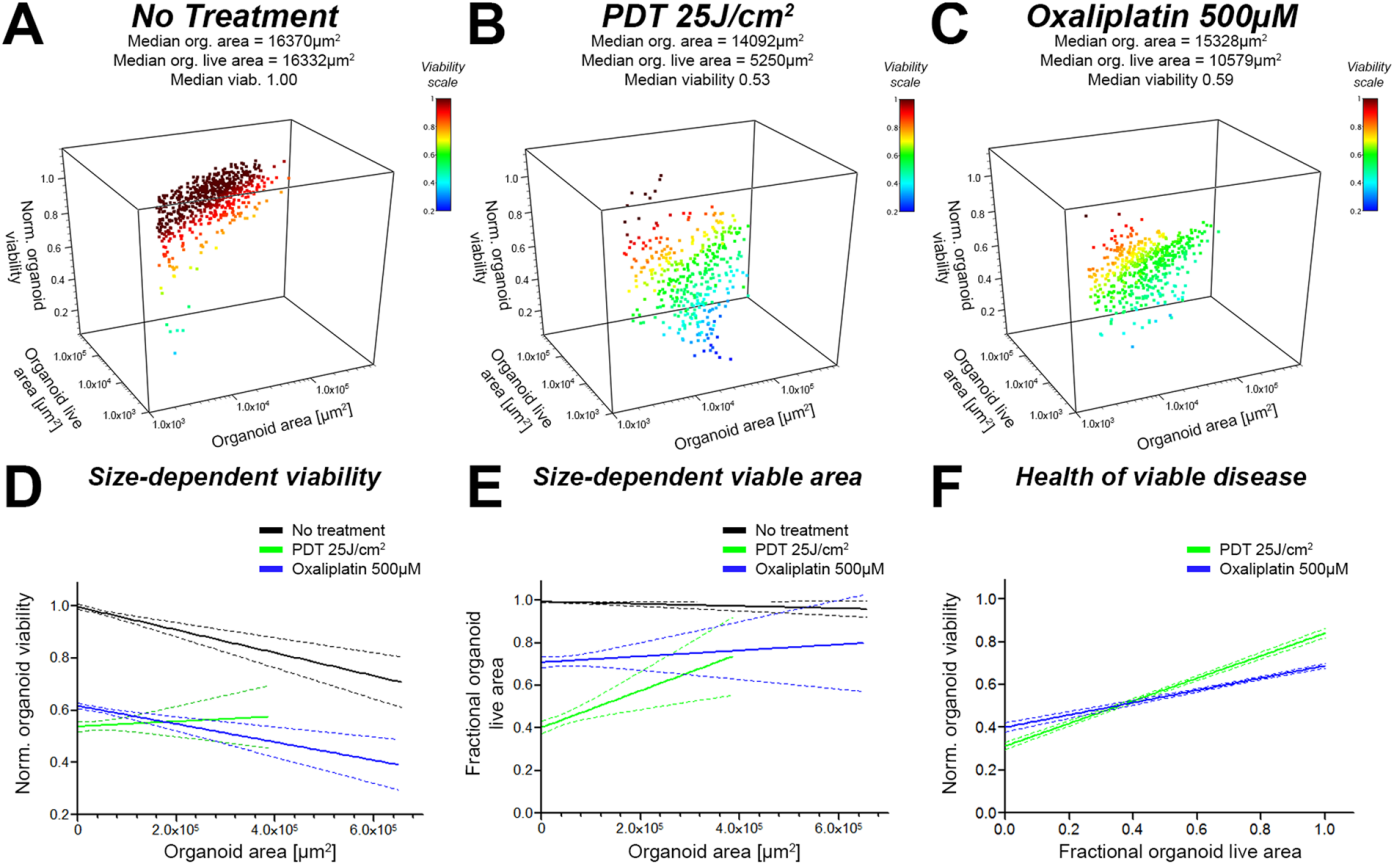
Readouts from CALYPSO are paired and allow advanced analysis of treatment response to investigate correlations between total residual area, residual viable area, and viability for every individual tumor organoid. Depicted here are results obtained on organoids made of MIA PaCa-2 cocultured with human dermal fibroblasts. Organoids were either untreated (black) or treated with either PDT at a radiant exposure of 25 J/cm^2^ (green) or 500 µM oxaliplatin (blue). Panels A, B and C represent 3D scatter plots of the paired data (organoid total area, organoid live are and viability) measured on cultures from (**A**) the no treatment control group, (**B**) the PDT (25 J/cm^2^) group, and (**C**) the 500 μM oxaliplatin group (72 h). Panels D, E and F represent linear regression performed on series of paired data allowing a deeper comparison of the effect of PDT and oxaliplatin chemotherapy regarding the no treatment control group. (**D**) Linear regression analysis between organoid viability and organoid size (area), displaying that PDT is more efficient in eradicating smaller organoids, whereas no size-related effects regarding viability are observed for oxaliplatin chemotherapy. (**E**) Linear regression analysis between fractional organoid live area and total organoid area, demonstrating that PDT reduces the relative live area more effectively for smaller organoids compared to larger organoids. (**F**) Linear regression analysis between organoid viability and fractional live area, demonstrating that PDT treatment leaves behind large viable nodules with high viability and small viable nodules with low viability. A similar trend was observed for oxaliplatin chemotherapy, although the steepness of the correlation suggests a much more homogeneous dispersion of viability and viable organoid size.

Cumulatively, the results depicted in Figs 3 and 4 illustrate that the single readouts display moderately dissimilar outcomes that individually do not fully capture the scope of treatment effects. A major advantage of CALYPSO is that all readouts are linked to the object index allowing multiparametric analysis to identify potential correlations between these metrics. In Fig. 5, these advanced correlations are exemplified using a similar coculture as previously described of MIA PaCa-2 and human dermal fibroblasts. Two treatment regimens that induce comparable median viabilities were chosen: BPD-PDT (25 J/cm^2^) and oxaliplatin (500 μM, 72 h) (Fig. S4). Despite having comparable median viabilities, there was a significant difference in the median fractional live area between the two treatment groups (Fig. S4E). A direct comparison of these readouts on a nodule-by-nodule basis may aid in understanding the origin of these discrepancies.

When plotting the viability of each individual tumor organoid relative to its total area, the differences between PDT and oxaliplatin become more apparent (Fig. S4B and C, respectively). When all data points were subjected to a linear correlation fit (Fig. S4D), the data suggests that the viability reduces slightly as a function of organoid size for both the no treatment control group and the oxaliplatin treated group. Conversely, a size-dependent increase in viability can be appreciated in the PDT-treated group. The size-dependent decrease in viability for the no treatment group is consistent with the oxygen diffusion limitations throughout bulky tumor organoids, creating a hypoxic core that accounts for a relatively larger area in the individual tumor nodules. The observation of a similar size-dependent viability correlation in the oxaliplatin-treated group in comparison to the no treatment group indicates that oxaliplatin exerts its effects homogeneously and independent of organoid size. This is in stark contrast with the PDT treated group, where the organoid size is positively correlated to viability, compellingly demonstrating that large tumor organoids are relatively less susceptible to PDT than their smaller counterparts. A similar trend is observed when comparing the total object area to the object’s fractional viable area (Fig. S4F and G). Upon fitting the data using a linear regression function, a slightly positive yet highly variable correlation between organoid size and the fractional live area was observed for oxaliplatin chemotherapy. A much steeper positive size-dependent live area correlation was found for PDT.

As viability is a metric that informs the health of the organoids independently of size and quantity of the viable residual disease, and that the fractional live area provides information on the amount of viable disease independent of viability, a direct comparison of these metrics may be useful in assessing the health of the residual viable disease. The scatter plots depicted in Fig. 5A,B and C depict a clear correlation between viability and fractional live areas after PDT and oxaliplatin chemotherapy, respectively. Linear correlation fits of the data (Fig. 5D–F) suggest that PDT on these cultures leaves behind a range of viable nodules. Amongst them the nodules presenting a fractional live area <0.5 have a substantially poorer health than nodules with a high fractional live area (>0.5) Fig. 5F. Contrary to PDT, oxaliplatin chemotherapy leaves behind a population of viable organoids that present a more homogeneous viability.

Together with the examples of live/dead fluorescence images depicted in Figs 3 and 4, these comparisons suggest that despite equal median viabilities, oxaliplatin chemotherapy yields a high amount of residual organoids of which the live signal emanates from the entire organoid area, whereas the live area of the PDT-treated organoids is more drastically reduced, albeit in a size-dependent manner. This corroborates the notion that PDT may be an effective debulking strategy, capable of reducing tumor size and effectively eradicating small tumor masses in the process.

Taken together, the multitude of readouts derived through the CALYPSO image analysis collaboratively provide a deeper understanding of treatment effects in terms of viability, size, and architecture of the remaining organotypic cultures. In addition, the image analysis workflow works well on heterotypic tumor cultures in which the individual tumor nodules were grown into complex geometrical shapes and heterogeneous sizes.

The comprehensive assessment of mesoscopic treatment effects may prove to be useful in therapy screening on patient derived tissue to guide treatment selection in the clinic^46^. For example, patient-derived tissues have been used to establish 3D histocultures in collagen sponge-matrices, which retained clinical relevance with higher architectural complexity than Matrigel cultures^47,48^. These histocultures were used for screening of therapeutics, wherein cytotoxicity was measured using an adapted MTT assay, and drug responses in the histocultures largely corroborated the observed drug responses in patients^49–52^. However, the MTT assay provides a single metric of treatment response, and a more comprehensive treatment response analysis may be informative when assessing mechanically distinct therapies or combination regimens, as was demonstrated in this study for chemotherapy, RT, and PDT. The live/dead staining combined with the CALYPSO image analysis is capable of extracting cell viability with spatial resolution, and may prove to be useful for application in patient-derived histocultures, such as the architecturally complex sponge-matrix cultures. Although such cultures were not explored in this study, there are no intrinsic limitations to adapt the live/dead staining and CALYPSO image analysis methodology for these culture types, although including spatial resolution on the z-axis could be of substantial additional value.

With respect to recapitulating human disease, all preclinical models have limitations: *in vivo* animal models are considered the most robust, but are expensive and too slow for rapid assessment of therapeutic efficacy. Moreover, they fail to incorporate human stromal components. The 2D cultures models are too simplistic to represent the architectural and biological complexities of cancers. The 3D models would appear to be an acceptable bridge and, as these evolve, we postulate that a multifaceted but relatively accessible analysis of treatment response is essential to fully comprehend various mesoscopic treatment effects.

A limitation of the live/dead staining and the CALYPSO image analysis procedure as presented here is that it only accounts for the live or dead status of the cells, but disregards biological aspects such as cell origin and dynamic heterogeneity (e.g., differentiation upon drug exposure). However, there is no intrinsic limitation in combining this live/dead analysis with pre-labeling of cells with dyes or genetically encoded fluorescent reporter proteins. This would bring additional information on cell type-based treatment susceptibilities provided that there is no overlap in emission spectra of the dyes. The CALYPSO image analysis workflow may be easily expanded to capture these intricate phenomena, although this remains to be further explored.

## Conclusion

The growing understanding of biological complexities underlying cancer warrants comprehensive preclinical models to expedite drug and therapy development, as well as their clinical translation. Our study has outlined a feasible image analysis workflow that is optimized to analyze a comprehensive scope of treatment effects on non-spherical organoid cultures of cancer, but which may similarly be applied to a wider range of organotypic cultures of human tissues, including patient-derived tissue explants. The results demonstrate the capacity of this approach to derive multiparametric dose responses of various, mechanistically distinct anticancer therapies and combinations thereof. Furthermore, CALYPSO can be utilized to identify the architectural effects of such therapies on both conventional and heterotypic adherent and suspended 3D cultures of cancer. As the methodology employs commercially available materials and widely accessible confocal fluorescence microscopy, the image analysis workflow described in this study has the potential for widespread implementation. The image analysis workflow can potentially be adapted to investigate more complex biological effects of the drug, e.g., cell-type dependent differences in treatment susceptibility and treatment-driven differentiation. As such, it presents as a valuable tool in the assessment and refinement of treatment strategies and may guide the design and translation of novel cancer therapies.

## Methods

### Cell culture and reagents

MIA PaCa-2 and AsPC-1 were obtained from the American Type Culture Collection, and NIH:OVCAR-5 cells were obtained from Thomas Hamilton (Fox Chase Cancer Institute, Philadelphia, PA). MIA PaCa-2 were cultured in Dulbecco’s Modified Eagle’s medium (DMEM), AsPC-1 and OVCAR-5 were maintained in Roswell Park Memorial Institute 1640 (RPMI) medium. All medium were supplemented with 1% (v/v) penicillin and streptomycin (Corning) and 10% (v/v) fetal bovine serum (FBS, Gibco). Primary dermal fibroblasts isolated from abdominal skin were kindly provided to us by Dr. Sandro Goruppi and prof. Gian Paolo Dotto (Cutaneous Biology Research Center, Massachusetts General Hospital and Harvard Medical School, Charlestown, MA). Fibroblasts were maintained in DMEM supplemented with 1% (v/v) penicillin, streptomycin, and 10% (v/v) FBS. All cell lines were maintained at standard culture conditions (37 °C, 5% CO2, 95% air).

### Adherent 3D cocultures

A 250 µL layer of 4 °C Matrigel was solidified in black walled glass bottom 24-wells plates (Corning) during a 20 min incubation at 37 °C. Subsequently, 7.5 * 10^3^ MIA PaCa-2, AsPC-1, or OVCAR-5 cells/well were seeded onto the Matrigel on day 1. Culture medium for 3D cultures was supplemented with 2% Matrigel throughout all experimental procedures, and cultures media was refreshed every 3/4 days.

For the cocultures, MIA PaCa-2 cells were seeded on day 1 as described above. On day 8, 5 * 10^4^ fibroblasts were added to the cultures, roughly corresponding to a MIA PaCa-2:fibroblasts ratio of 1:1. The cocultures were cultured until day 11, after which therapy was initiated (see below). Experiments were terminated on day 15.

### Suspension 3D cultures

Suspended spheroids were cultured in 96 well, round bottom ultra low attachment plates (Corning Costar, Kennebunk, ME). AsPC-1 cells were seeded on day 1 at a density of 2.5 * 10^3^ cells per well in a volume of 100 µL. PDT was initiated on day 3 by adding 100 µL of 0.5 µM BPD to the culture medium (final concentration 0.25 µM BPD). After 1 h of incubation with BPD, the organoids were irradiated with 690 nm laser light (150 mW/cm^2^, 1–80 J/cm^2^). The experiment was ended on culture day 5, on which viability was assessed.

### Treatments on adherent 3D cultures

For adherent 3D cultures, oxaliplatin chemotherapy (Selleckchem, Houston, TX) was given at the indicated concentrations (100–1000 µM). Cultures were exposed for 72 h from day 11 to day 14, after which culture medium was refreshed and cultures were maintained for another day.

For PDT, cells received fresh culture medium supplemented with 0.25 µM verteporfin (benzoporphyrin derivative, BPD, Sigma-Aldrich, St. Louis, MO). After 60 min of incubation with BPD, the culture media was refreshed and cultures were irradiated at a fluence rate of 150 mW/cm^2^, 690 nm laser light (Intense, North Brunswick, NJ), at the indicated radiant exposures (1–50 J/cm^2^). In line with the oxaliplatin treatment groups, PDT-treated cultures received fresh culture media on day 14.

Cultures received fresh medium before receiving X-ray radiation therapy (RT). RT was delivered by an X-rad 320 source that operates at 320 kV and 12.5 mA. A total dose of 10 Gy was delivered at a rate of 2.75 Gy/min through a 2 mm aluminum filter.

### Live/dead imaging

The treatment outcomes were evaluated by staining the live and dead cell populations *in situ*. Two internal control groups were essential for this assay: a no treatment and a total killing (TK) control group. TK was achieved by a two-step process. First, cells were fixed during a 5 min incubation with a 4% formalin solution in PBS (with Ca^2+^ and Mg^2+^, Gibco) after which membrane permeabilization was performed by a 30 min incubation with 0.1% Triton X-100 (Sigma-Aldrich). Following Triton X-100 incubation, the TK wells were washed thrice with 0.1 M Glycine (Sigma-Aldrich). Subsequently, all wells received 500 μL of PBS supplemented with 2 µM calcein AM (ThermoFisher) and 3 µM PI (ThermoFisher). For suspension cultures, 100 μL of PBS supplemented with 4 μM calcein AM and 6 μM PI was added directly to the 100 μL of culture medium. Cultures were incubated for 30 min at standard culture conditions prior to imaging.

Fluorescence signals were recorded using an Olympus FV-1000 confocal microscope through a 0.16NA 4x air objective. In each of the 24 wells, a mosaic composed of four images (512 * 512px) was acquired; covering a total field of view of approximately 6.4mm^2^ in the central area of every well. At the expense of axial resolution, the fluorescence signal was maximized by setting the confocal pinhole at 500 µm. The axial position of the imaged plane was chosen to maximize the amount of signal collected. The scanning time was 4 µs/pixel. Fluorescence intensities were acquired at λex = 488 nm/λem = 520 ± 20 nm (calcein) and λex = 559 nm/λem = 630 ± 20 nm (PI). Brightfield images were acquired under 559 nm light.

### Image analysis

Image analysis was performed using a custom-built workflow in Matlab 2016b (Mathworks, Natick, MA). The code comprised four main components:

#### Masking

The mask outlining the culture objects was derived from the brightfield image by calculating an adaptive threshold set at a sensitivity of 0.55. The threshold was calculated by integrating the mean intensity over a neighborhood size equal to 65 * 65px for 512 * 512px resolution. Subsequently, a mask was created by binarizing the brightfield image using the adaptive threshold. A size threshold was set at 1950 µm^2^; identifying individual objects greater than 5 clustered cells (of 400 μm^2^) as tumor organoids of interest. To exclude objects that were out-of-focus, the live and dead channels were summed, converted to grayscale (ranging from 0 to 1), and binarized using Otsu’s thresholding method. The mask and the binarized fluorescence images were multiplied to provide the final mask. The final mask and the nodule indices were subsequently applied to both fluorescence channels.

#### Background subtraction

To limit the role of the cell debris or detached cells in the quantification of the background intensity, images from the no treatment group were used and the mask was slightly dilated, using a disk shaped structuring element with a radius of 2px. The median pixel fluorescence intensities (calcein and PI) of the individual reverse-masked images were averaged, which were then subtracted from their respective channels for all images in the individual experiments.

#### Threshold calculation

To extract the residual live area of each nodule, the calcein AM fluorescence intensity was binarized for quantification of the absolute live area and fractional live area of the tumor organoids. For this step, a fixed threshold value was applied to all images. The threshold value was obtained by applying Otsu’s method to the no treatment group, which was multiplied by a factor of 0.4 to facilitate optimal analysis of our images, *i*.*e*., to set up the dynamic range for the fractional live area between 0 (based on TK controls) and 1 (based on no treatment controls). For each nodule, the amount of pixels exceeding the threshold was summed to provide the area of the live residual disease per tumor organoid.

#### Nodule-by-nodule analysis of size, viability and thresholded live area

The customized Matlab routine gives on a nodule-by-nodule fashion the organoid size (in μm^2^), the residual and fraction live area as well as the viability allowing a correlated analysis of these parameters. Total organoid area was computed by multiplying the thresholded area (in px) by the area/px (in µm^2^). Organoid viability was calculated using the live/dead fluorescence intensities as described in the Results and Discussion, and were typically normalized to the no treatment control groups. Organoid fractional live area was calculated by dividing the organoid’s thresholded live area by the organoid’s total area (brightfield).

### Statistical analysis

All data was statistically analyzed in Graphpad Prism 5.0 (La Jolla, CA). Data sets were tested for Gaussian distributions using a D’Agostino and Pearson omnibus normality test. Normally distributed data sets were analyzed using a One-way ANOVA and Bonferroni post-hoc test for multiple comparisons, whereas non-Gaussian data sets were analyzed using a Kruskal-Wallis and Dunn’s post-hoc test for multiple comparisons. Statistical significance is indicated with single asterisks (p = < 0.05), double asterisks (p = < 0.01) or triple asterisks (p = < 0.005). Dose-response curves were fitted whenever appropriate using the least-squares fit, and is typically depicted as a red line in the corresponding graphs.

## Electronic supplementary material

Supplementary Information
