# Supplementary material for: Comprehensive high-throughput image analysis for therapeutic efficacy of architecturally complex heterotypic organoids
Source: Sci Rep. 2017 Nov 30;7:16645. doi: 10.1038/s41598-017-16622-9 (PMC5709388; doi:10.1038/s41598-017-16622-9)
Supplement: Supplementary file 1 — Supplementary Information [file 41598_2017_16622_MOESM1_ESM.pdf]

SUPPLEMENTAL INFORMATION

Comprehensive high-throughput image analysis for therapeutic efficacy of architecturally complex heterotypic organoids

Anne-Laure Bulin[#], Mans Broekgaarden[#], and Tayyaba Hasan[*]

Wellman Center for Photomedicine, Department of Dermatology, Harvard Medical School and Massachusetts General Hospital, 40 Blossom Street, 02114 Boston, MA.

thasan@mgh.harvard.edu

Figure S1

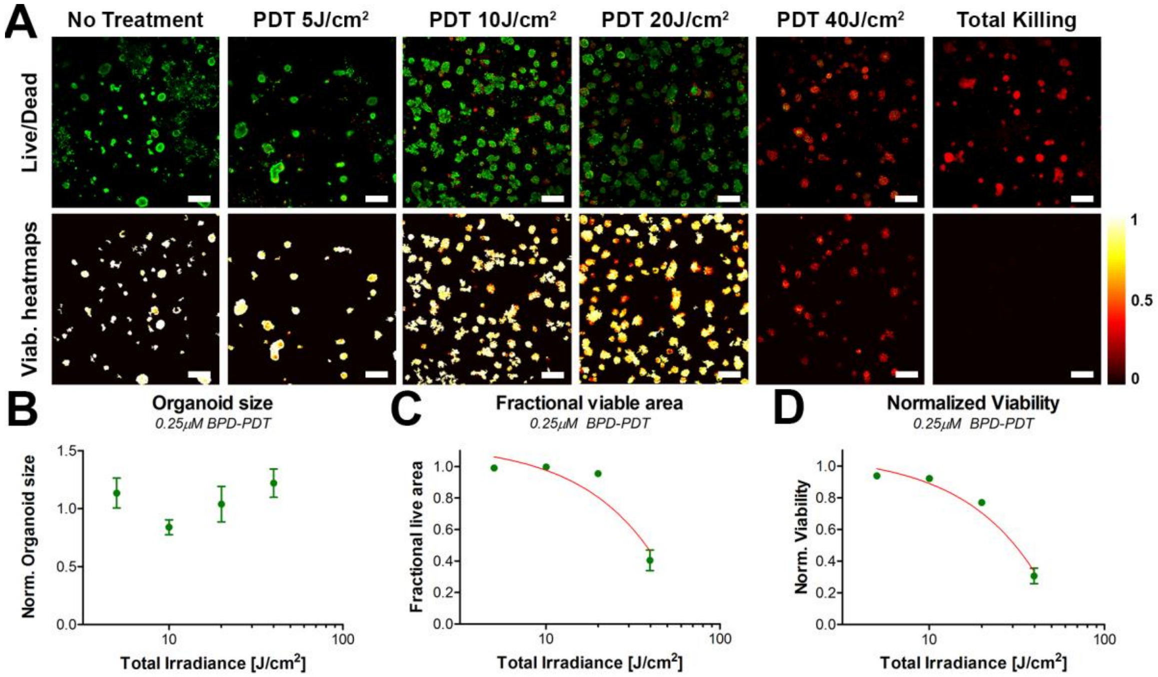

Figure S1: Dose response relation between the total light irradiance of PDT and organoid size, fractional viable area, and normalized viability on OVCAR-5 human ovarian carcinoma cells grown as 3D adherent cultures. (A) Live/dead images of calcein and PI fluorescence were superimposed in ImageJ and depicted side-by-side with the corresponding viability heatmaps. Scalebar = 400µm. (**B**-**D**) Dose response correlations between the PDT radiant exposure and the total organoid area (mean ± SEM) (**B**), median fractional live area (mean ± SEM) (**C**), and the median viability of the tumor nodules (mean ± SEM) (**C**). Data represents three replicate experiments in which N = 24-36.

Figure S2

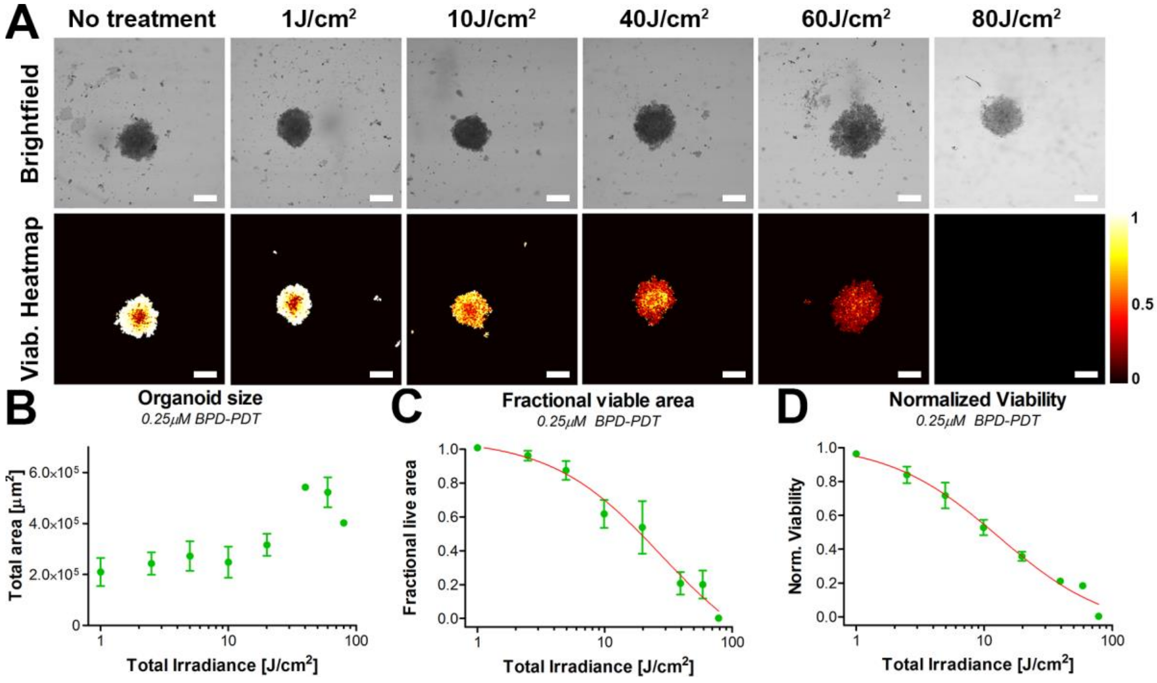

Figure S2: Primary output parameters obtained through CALYPSO enables to report the effect of PDT on AsPC-1 spheroids grown as monoculture in suspension. (**A**) Representative brightfield images of the nodules are depicted side-by-side with the corresponding viability heatmaps for increasing doses of PDT. (**B-D**) Dose response correlations between the PDT radiant exposure and the total organoid area (mean ± SEM) (**B**), median fractional live area (mean ± SEM) (**C**), and the median viability of the tumor nodules (mean ± SEM) (**C**). Data represents a single representative experiment (N = 4).

Figure S3

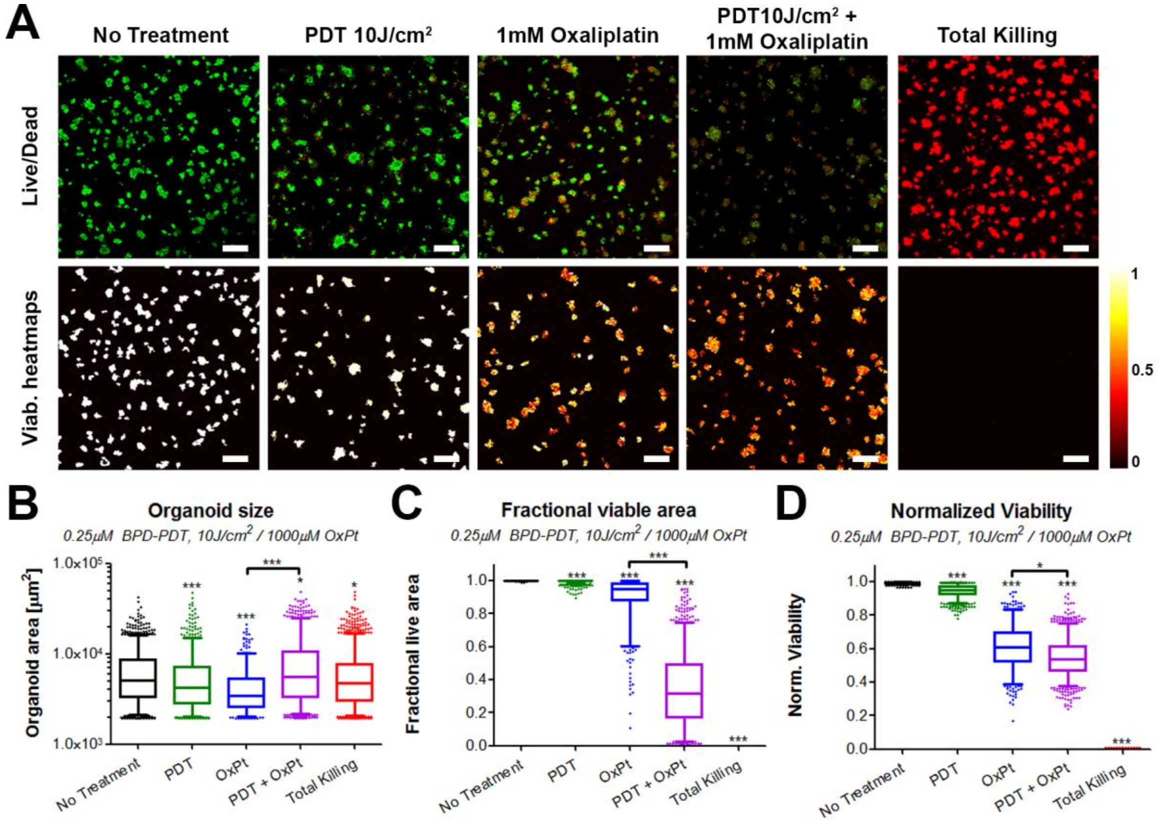

Figure S3: Analysis of treatment effects on 3D adherent AsPC-1 cultures following PDT, Oxaliplatin (OxPt) chemotherapy, or a combination therapy consisting of PDT with subsequent OxPt. (**A**) Live/dead images of calcein and PI fluorescence were superimposed in ImageJ and depicted side-by-side with the corresponding viability heatmaps. Scalebar = 400μm. (**B-D**) Box-whisker plots depicting the spread of the data pertaining to the organoid size, fractional viable area, and normalized viability of the AsPC-1 organoids following treatment with either PDT (green), OxPt (blue), or a combination therapy of PDT+OxPt (purple). No treatment controls and total killing controls are depicted in black and red, respectively. All box-whisker plots depict the median, 25th and 75th percentile, the 95% confidence interval, and the outliers, which were extracted and depicted on a nodule-by-nodule basis. Data was obtained from a single representative experiment comprising a sample size of ~800-1200 tumor organoids per condition.

Figure S4

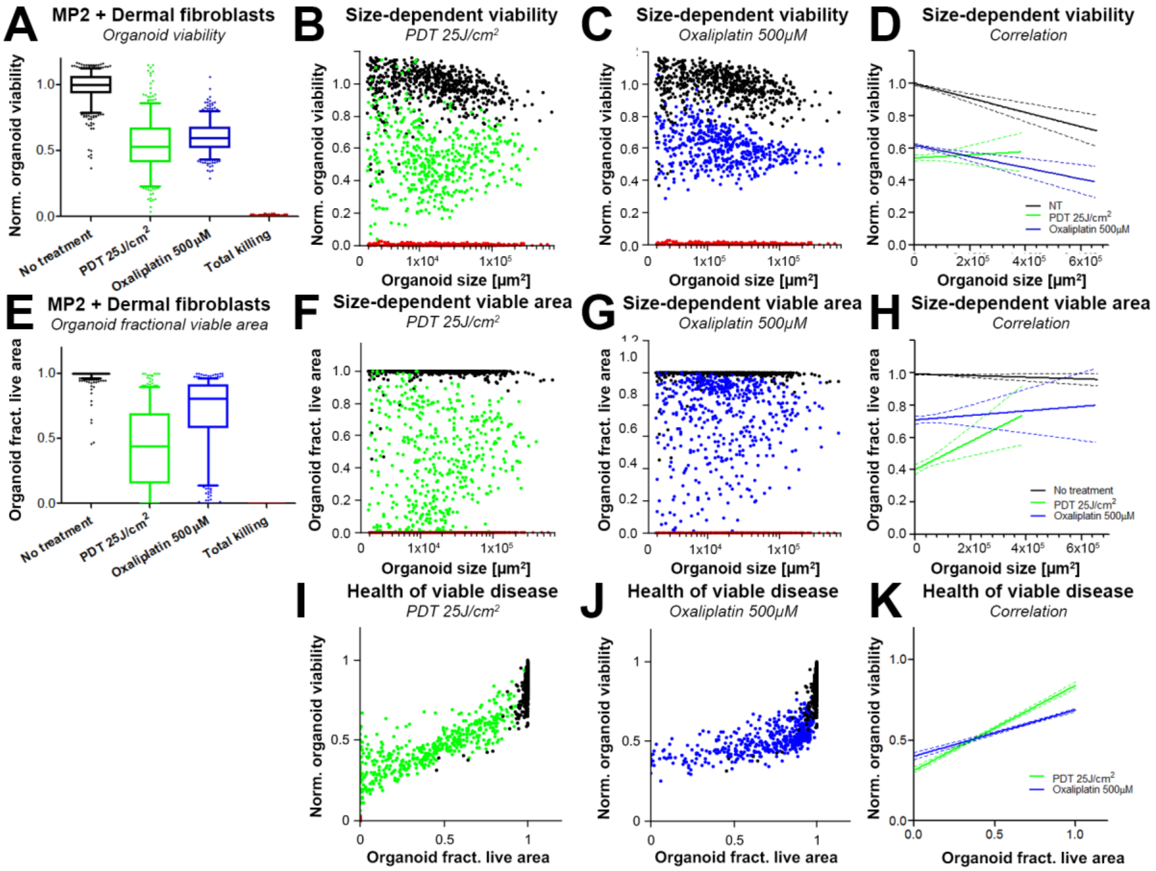

Figure S4: The spread of the individual organoid viabilities following treatment is depicted in (**A**), displaying the median, 25th and 75th percentile, and the 95% confidence interval. Organoid viabilities plotted against the organoid size (total area) are depicted in panels (**B**) and (**C**), for which the linear fits ± 95% CI are plotted in (**D**). The spread in fractional live area of the individual organoid following treatment is depicted in (**C**), displaying the median, 25th and 75th percentile, and the 95% confidence interval. Fractional live areas are plotted against the organoid size (total area) in panels (F) and (**G**), for which the linear fits ± 95% CI are plotted in (**H**). The individual organoid viability as a function of fractional live area after PDT and chemotherapy are plotted in panels (**I**) and (**J**), for which the linear correlation fits are plotted in panel (**K**).
